# Dietary pattern and precocious puberty risk in Chinese girls: a case-control study

**DOI:** 10.1186/s12937-024-00916-6

**Published:** 2024-01-31

**Authors:** Qiuyun Gu, Youmei Wu, Zhuowei Feng, Yimeng Chai, Shan Hou, Zhiping Yu, Xiuhua Shen

**Affiliations:** 1 Department of Nutrition, Shanghai General Hospital, Shanghai Jiao Tong University School of Medicine, Shanghai, China; 2 Department of Clinical Nutrition, College of Health Science and Technology, Shanghai Jiao Tong University School of Medicine, Shanghai, China; 3 Shanghai Key Laboratory of Pediatric Gastroenterology and Nutrition, Xinhua Hospital, Shanghai Jiao Tong University School of Medicine, Shanghai, China; 4 Department of Statistics, Cancer Hospital of the University of Chinese Academy of Sciences, Zhejiang, China; 5 Department of Paediatrics, Xinhua Hospital, Shanghai Jiao Tong University School of Medicine, Shanghai, China; 6 Department of Nutrition and Dietetics, Brooks College of Health, University of North Florida, Jacksonville, FL USA; 7 Department of Nutrition, School of Public Health, Shanghai Jiao Tong University, Shanghai, China; 8 Department of Clinical Nutrition, Xinhua Hospital, Shanghai Jiao Tong University School of Medicine, Shanghai, China

**Keywords:** Precocious puberty, Dietary intake, Case-control study, Fruits, Vegetables, Red meat, Girls

## Abstract

**Background:**

The role of dietary intake on precocious puberty remains unclear. This study aimed to investigate the association between the amount and frequency of dietary intake and the risk of precocious puberty in Chinese girls.

**Methods:**

In this case-control study, we enrolled 185 precocious puberty girls and 185 age-matched controls. Their dietary intake was assessed through a semi-quantitative food frequency questionnaire. Their sociodemographic and lifestyle data were collected. The associations between dietary intake and risk of precocious puberty were assessed by conditional logistic regression models.

**Results:**

After multivariate adjustment, consuming a higher amount of red meat was associated with higher precocious puberty risk (OR = 2.74, 95% CI: 1.25–6.02), while a higher frequency of fruit ( *P* for trend = 0.024) and amount of vegetable intake was associated with a lower risk of precocious puberty (*P* for trend = 0.002). The high vegetable and protein dietary pattern was significantly negatively associated with precocious puberty (OR = 0.78, 95% CI: 0.63–0.97), whereas the high animal food and fruits dietary pattern was remarkably positively associated with precocious puberty (OR = 1.36, 95% CI: 1.09–1.69), after adjusting for age and body mass index.

**Conclusions:**

High vegetable and protein dietary pattern is a protective factor against precocious puberty, while high animal food and fruits dietary pattern is a risk factor for precocious puberty in Chinese girls. Attentions should be paid to a reasonable intake of red meat, eggs, and fruits in children’s daily diet, increase their intake of vegetables, in order to reduce the risk of precocious puberty.

**Supplementary Information:**

The online version contains supplementary material available at 10.1186/s12937-024-00916-6.

## Introduction

Precocious puberty refers to a phenomenon in which boys show secondary sexual characteristics before the age of 9 and girls before the age of 8 [1]. Based on pathogenesis, it can be divided into Central Precocious Puberty (CPP) and Peripheral Precocious Puberty (PPP) [2]. Reports indicate that the global incidence of precocious puberty in children is about 1 in 5000 to10000 with a male-to-female ratio of approximately 1:3 to 1:23 [3]. Moreover, the proportion of children suffering from idiopathic CPP has increased greatly in many countries in recent years [4]. For example, the incidence ranges from around 0.38–0.74% depending on the survey area in China [5]. Notably, the incidence of the condition in girls is higher than in boys [5]. In South Korea, the incidence of CPP rose from 89.4 to 415.3 per 100,000 in girls under 9 years from 2008 to 2014 [6]. Precocious puberty in children can lead to short stature in adulthood [7]. Moreover, many studies have shown that girls with precocious puberty are at a high risk of cardiovascular diseases, diabetes, and breast cancer once they become adults [8, 9]. In addition, girls with precocious puberty are more likely to have psychological and behavioral problems during adolescence [10, 11].

Dietary intake may play a significant role in pubertal timing [12]. However, due to different study populations and dietary assessment methods, the evidence to date was not consistent regarding the association between dietary intake and the onset of puberty. For instance, our prior study found that higher intake of poultry was associated with an earlier age at menarche, while neither the intake of pork, beef, lamb, processed meat nor total meat intake was associated with menarche age in Chinese girls [13]. Carwile et al. reported no association between peri-pubertal meat intake and age at menarche in 5583 girls from the United States [14]. Previous research suggests that increased intake of vegetables and fruits may delay pubertal development. Two UK cohort studies found that higher vegetable protein intake corresponded with later pubertal growth spurt, peak height velocity, and age at menarche [4, 15]. A prospective study of children from Mexico City revealed that a dietary pattern rich in vegetables and lean meats was associated with delays in breast development, while a dietary pattern rich in fruits and yogurt was not statistically significantly associated with any of the sexual maturation markers [16]. Likewise, the influence of the frequency of milk intake on early menarche was also controversial. In a study of 1,008 American girls, it was shown that the frequency of milk intake in girls between 5 and 12 years was negatively correlated with their age at menarche [17]. Nevertheless, a prospective cohort study found that the frequency of milk intake could not predict age at the onset of menarche [14]. These findings suggest that childhood diet may be one of the controllable risk factors for central precocious puberty. The previous studies primarily focused on children’s dietary frequency and pattern and precocious puberty [13, 18–20], leaving gaps in understanding the relationship between the frequency as well as amount of dietary intake and precocious puberty in Chinese girls. Besides, these studies lacked the analysis of the relationship between seven foods recommended by dietary guidelines for Chinese school-aged children issued in 2022 [21].

In this study, we hypothesize that dietary patterns were related to precocious puberty among Chinese girls. The dietary frequency and intake of 185 girls with precocious puberty and 185 healthy girls were investigated, and the dietary patterns of children were assessed by principal component analysis to explore the relationship between dietary patterns and precocious puberty. Furthermore, this study aimed to investigate the association between precocious puberty and the frequency as well as amount of dietary intake in a paired case-control design in Chinese girls. Additionally, this research was the first one to analyze the relationship between seven foods recommended by dietary guidelines for Chinese school-aged children issued in 2022 [21] and precocious puberty in Chinese girls. The findings will provide valuable evidence for developing preventive dietary guidance for girls.

## Methods

### Sample size calculating

This study employed a 1:1 paired case-control research design, with pairing based on age. The required sample size was calculated using the sample estimation formula for a case-control study with a 1:1 matching design (Formula 1).

1 $$M=\frac{[{Z}_{\alpha }/2+{Z}_{\beta }\ast \sqrt{P(1-P)}]/(P-\frac{1}{2})^2}{{P}_{0}\ast (1-{P}_{1})+{P}_{1}\ast (1-{P}_{0})}$$

In Formula 1, M represents the total number of pairs, ${Z}_{\alpha }$and${ Z}_{\beta }$ are the critical values corresponding to the probabilities $\alpha$ and $\beta$ in the standard normal distribution, respectively. ${P}_{0}$ and ${P}_{1}$ denote the estimated exposure rates of a specific exposure factor in the control and case groups, respectively.

Taking the consumption of poultry and livestock as one of the outcome indicators, based on existing literature [22], the exposure rate in the control group (${P}_{0}$) for high poultry and livestock intake was estimated to be 0.56, with an odds ratio (OR) of 2.50. Choosing a significance level ($\alpha$) of 0.05 (two-sided) and a power ($\beta$) of 0.10, the corresponding critical values were${Z}_{\alpha }/2$ = 1.96 and ${Z}_{\beta }$ = 1.282. Subsequent calculations yielded M = 118, and we finally enrolled participants were 185 pairs.

### Study design and study population

In this case-control study, we recruited 468 participants aged 5–12 years between March 2017 and August 2019 in the Department of Pediatric Endocrinology, Xinhua Hospital Affiliated to Shanghai Jiao Tong University School of Medicine. Among them, 408 girls (204 precocious puberty girls, 204 aged-matched controls) agreed to complete the questionnaire. A total of 38 girls were excluded for either incomplete or implausible information. Consequently, 185 precocious puberty girls and 185 age-matched controls were included in the analysis.

### Selection of cases and controls

The case group was recruited girls diagnosed with precocious puberty. The diagnostic criteria for precocious puberty were as follows: (1) the development of secondary sexual characteristics (breast growth [23, 24]) before 8 years of chronologic age or menarche before 10 years of chronologic age; (2) advanced bone ages ≥ 1 year above the chronologic age; (3) LH peak ≥ 5 IU/L at the GnRH stimulation test [25]. (4) Transpelvic B ultrasound showed the presence of enlarged uterine and ovarian volumes, and multiple follicles with a diameter of more than 4 mm; (5) Pituitary MRI showed no organic lesions [26]. The case group excluded (1) girls with precocious puberty for a clear cause due to incorrect contraceptive use; (2) Precocious puberty caused by organic lesions such as ovarian tumors and hypothalamic hamartomas; (3) Suffering from any endocrine system disease or chronic kidney disease, epilepsy and other chronic diseases [27]. As for assessing breast growth, the development of the breasts was clinically scored on a scale from 1 to 5 (B1–B5) according to the method described by Tanner [23], which is based on a visual inspection, although palpation of the breast was included in this procedure. Pubertal onset is defined as Tanner B2, which is characterized by the visual and/or palpable appearance of glandular tissue [24].

The inclusion criteria for the control group were as follows: 1). Healthy girls who were not diagnosed with precocious puberty in Xinhua Hospital and whose ages matched the case group. 2). Girls did not reveal the development of secondary sexual characteristics. However, those with central system, endocrine, reproductive system, thyroid system, adrenal system, or digestive system diseases were excluded.

### Anthropometric indices and dietary assessment

Information on the demographic and lifestyle data of participants in both groups was collected through face-to-face interviews to children’s parents by ten trained dietitians using a structured questionnaire in the Department of Pediatric Endocrinology, Xinhua Hospital Affiliated to Shanghai Jiao Tong University School of Medicine. This included age, physical activity (referring to moderate-to-vigorous exercise time for extracurricular physical activities such as cycling, running, swimming, dancing, and team sports [28]), sleep pattern, dietary habits, questions on parents, and questions on the usage of adult toiletries (such as cosmetics, body wash, and skin cleanser) and homework (consists of tasks that teachers assign students to perform at home). Whether children slept with light exposure at night was categorized as “yes” or “no”. Additionally, whether children felt a heavy homework burden was categorized as “yes” or “no”. Dietary habits were dichotomized into the categories according to the recommendations on “Student Meal Nutrition Guidelines” were issued by China’s Health and Family Planning Commission in 2017 [29], “Meat and vegetable balance” [referring to eating meat (= 80 g/d) and vegetables (= 350 g/d) per day], “More meat and less vegetable “[referring to eating more meat (> 80 g/d) and less vegetables (< 350 g/d) per day], “More vegetable and less meat” [referring to eating more vegetable (> 350 g/d) and less meat (< 80 g/d) per day] and “Others” (referring to eating habits other than the three above). The continuous variables (such as the father’s age at first spermatorrhea and the mother’s age at menarche) were recorded. Participants were asked to choose the answer that applied best to the situation in the last 12 months before the first visit to the hospital (Cases group: before the onset of precocious puberty). The questionnaire was completed by girls and their parents together.

As for sleep time, trained dietitians asked parents the following question: (a) “On average, how many hours do your children sleep per day?” Children were further grouped into 2 categories (< 8 h, and ≥ 8 h per day) based on their average daily sleep time.

Additionally, physical examination was done by trained dietitians to obtain the height and body weight of the participants, following a standardized protocol. Body mass index (BMI) was calculated as weight (kilograms) divided by the square of height (meters^2^).

A modified version of the simplified food frequency questionnaire (FFQ), which was derived from our previous research [30, 31], was employed to collect data on the frequency and intake of food during the preceding 12 months. The FFQ focused on the most commonly consumed foods that were deemed potentially associated with early onset puberty from a clinical perspective [18]. These foods included red meat, poultry, fish and shrimp, soy products, vegetables, fruits, dairy products, eggs, fried foods, and soft drinks, as presented in Table S1. Briefly, for each food item, participants and their guardians reported the frequency of habitual consumption (daily, weekly, monthly, annually, or never (the reference category)) and the amount consumed in the past 12 months (Case group: before the onset of precocious puberty). The assessment was conducted by a trained nutrition professional on a one-on-one basis. The high reliability and validity of FFQ for assessment of related food consumption was shown in certain studies [13, 30, 32].

### Dietary patterns analysis

Exploratory factor analysis was utilized to identify prominent dietary patterns, taking into account the outcomes of the Kaiser-Meyer-Olkin (KMO) and Bartlett’s test. The scree plot was employed to determine principal components, whereby factor loading values ≥ 0.3 or ≤ − 0.3 were deemed significant contribution thresholds to the pattern. Principal component analysis with varimax rotation was employed to compute the factor scores of each pattern for every individual. This study identified common factors based on food characteristics and professional expertise. Factor scores were computed, and participants were categorized into quartiles (Q1, Q2, Q3, and Q4) based on their respective factor scores. Logistic regression analysis, incorporating the factor score, was employed to evaluate the correlation between dietary patterns and precocious puberty [18].

### Statistical analysis

Statistical Package for Social Sciences (SPSS) version 25 was used for data analysis. The paired *t*-test was used to compare the difference of continuous data between the case and the control groups. The paired chi-square test was used to analyze categorical data between groups. The conditional logistic regression model was used to determine the OR of precocious puberty by tertiles of dietary variables after adjusting for confounding variables. The three rules we used to adjust for variables aimed to minimise potential confounding effects. In the final model, we adjusted for factors that met at least one of the following criteria:① We adjusted for variables (sleep with light exposure, use adult toiletries, heavy homework burden, and dietary habits, mother’s age at menarche) with a *P*-value of less than 0.05 in the univariate analyses by including them in the multivariate analyses; ② In the multivariate analysis, we also selected variables based on previous findings and clinical constraints. These variables included physical activity, sleep time, father’s age at first spermatorrhea, family income, and parents’ educational level were further adjust [18, 33, 34]. According to the frequency distribution, the frequency and intake of each food were recoded into three or four categories to analyze the effect of a single food on precocious puberty.We performed tests for linear trends with the use of tertiles of the dietary variable as a continuous variable by assigning the median values of the tertiles to the variable. Statistical significance was set at *P* < 0.05.

### Ethics statement

This study was carried out in accordance with the Declaration of Helsinki and was approved by the Institutional Review Board of the Department of Pediatric Endocrinology, Xinhua Hospital Affiliated to Shanghai Jiao Tong University School of Medicine (NCT03628937) [30]. Informed consent was obtained from guardians.

## Results

### Comparison of the general characteristics between the case group and the control group

The average age of girls in the case group and the control group was 7.84 ± 1.05 and 7.71 ± 1.48 years (*P* > 0.05), respectively (Table 1). Despite the higher weight and height of girls in the case group than in the control group, there was no statistical difference in BMI between the two groups (*P* = 0.09). The rate of sleeping with light exposure, use of adult toiletries, and percentage of participants claiming heavy homework burden was significantly higher in the case group than that in the control group (*P* < 0.05). The obesity rate of the case group was higher than that of the control group (*P* < 0.05). In addition, the percentage of participants having more meat and fewer vegetables was higher in the case group than in the control group. There were no significant differences in other characteristics between the two groups (Table 1).

**Table 1:** Comparison of the general characteristics between the case group and the control group

Characteristics^1^	Cases (*n* = 185)	Controls (*n* = 185)	*P* value
**Girls**			
Age, y	7.84 ± 1.05	7.71 ± 1.48	0.31
Height, cm	133.57 ± 6.91	130.87 ± 10.05	< 0.001
Weight, kg	30.30 ± 6.36	28.48 ± 6.96	0.006
BMI, kg/m^2^	17.03 ± 3.14	16.49 ± 2.99	0.09
Nutritional Assessment, N(%) ^2^			
Obesity	34 (18.38)	23 (12.43)	0.044
Overweight	32 (17.30)	30 (16.22)	
Normal	112 (60.54)	111 (60.00)	
Mildly emaciated	2 (1.08)	10 (5.41)	
Moderately to severely emaciated	5 (2.70)	11 (5.95)	
Heavy homework burden (Yes), %	31.52	17.39	0.002
Always live with mother (Yes), %			0.10
Yes	89.19	95.68	
No	10.81	4.32	
Always live with father (Yes), %			0.50
Yes	84.32	88.11	
No	15.68	11.89	
Dietary habits, %			< 0.001
Meat and vegetable balance	26.86	37.30	
More meat and less vegetable	58.86	38.92	
More vegetable and less meat	10.29	20.54	
Others	4.00	3.24	
**Mothers**			
Mother’s age at menarche, y	13.38 ± 1.32	13.76 ± 1.28	0.06
Age at girl’s birth, y	27.46 ± 3.95	27.87 ± 4.18	0.45
Illness during pregnancy (Yes), %	5.98	4.89	0.65
Illness during lactation (Yes), %	3.80	3.26	0.78
Exposure to toxic substances during pregnancy^3^ (Yes), %	7.61	4.35	0.22
Mother’s education level, %			0.26
Middle school or lower	9.19	11.41	
High school	18.38	12.50	
College or higher	72.43	76.09	
**Father’s**			
Father’s age at first spermatorrhea, y	13.08 ± 1.32	13.76 ± 1.28	0.46
Father’s education level, %			0.74
Middle school or lower	9.19	11.41	
High school	15.68	14.13	
College or higher	75.14	74.46	
Family income (Yuan/month), %			0.98
< 3000	1.66	1.70	
3000 ~ 5000	6.08	5.11	
5000 ~ 8000	16.02	15.34	
> 8000	76.24	77.84	

^1^Mean (standard deviation), unless otherwise stated.

^2^Nutritional assessment was evaluated by 《WS/T 586–2018 Screening for Overweight and Obesity in School-age children and Adolescents》 and 《WS/T 456–2014 Screening for malnutrition in school-age children and adolescents》.

^3^Toxic and harmful substances include chemical pollutants (gasoline, paint, leather, etc.), and pesticides, etc.

### The dietary composition of girls in the case and the control groups

In this study, the vegetable intake of girls in the case group (163.99 ± 122.61 g/day) was significantly lower than that of the control group (217.07 ± 147.54 g/day) (*P* < 0.05). There were no significant differences in the intakes of red meat and poultry, fish and shrimp, milk, eggs, and soy products between the two groups as shown in Table 2.

**Table 2:** The dietary composition of girls in the case and the control groups

Food parameter (g/d)	Cases (*n* = 185)	Controls (*n* = 185)	Recommended intake^3^ (g)
Red meat	46.95 ± 31.87	43.75 ± 41.26	
Poultry	12.58 ± 19.26	13.74 ± 17.35	
Red meat and poultry	59.53 ± 37.78^2^	57.49 ± 51.11^2^	40
Fish and shrimp	38.23 ± 44.60	38.99 ± 39.73	40
Vegetables	163.99 ± 122.61^1,2^	217.07 ± 147.54^2^	300
Fruits	185.98 ± 134.49	208.62 ± 121.94	175
Dairy products	206.22 ± 148.18^2^	208.97 ± 145.73^2^	300
Eggs	41.15 ± 33.70	48.75 ± 50.92^2^	40
Soy products	13.72 ± 24.45	13.94 ± 17.87	15

^1^Compared to the control group, *P* < 0.05

^2^Compared to the recommended intake, *P* < 0.05

^3^Recommended intake was from dietary guidelines for Chinese school-aged children issued in 2022

The “dietary guideline for Chinese school-aged children” was issued by Chinese Nutrition Society in 2022 [35]. The dietary intake of children in the case and the control groups were compared to the recommended intake for girls aged 6–10. In both groups, the intakes of poultry and red meat were higher than the recommended intake by diet pagoda (*P* < 0.05) while the intakes of vegetables and dairy products were lower than the recommended intake by diet pagoda (*P* < 0.05). In addition, in the case group,- only the consumption of eggs was higher than the recommended amount by diet pagoda (*P* < 0.05). No significant difference was found in terms of fruits, soy products, fish and shrimp when compared both groups with the recommended intake by diet pagoda .

### Frequency of dietary intake and precocious puberty risk

**Fig. 1:**
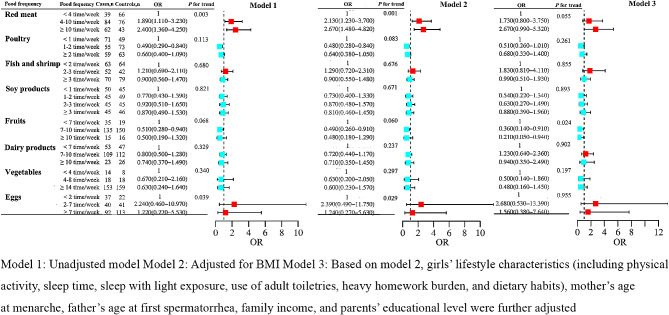
Odds ratios for precocious puberty risk across the frequency of dietary intake

Conditional logistic regression models were used to explore the effects of frequency of dietary intake on girls with precocious puberty (Fig. 1).

In model 3, after multivariate adjustment, higher fruit intake frequency was associated with a lower risk of precocious puberty. Specifically, the adjusted ORs (95% CI) for the risk of precocious puberty in the fruit intake of the 7–10 times/week group and 10 times/week group were 0.36 (0.14, 0.91) and 0.21 (0.05, 0.94), respectively compared to the < 7times/week group (*P* for trend = 0.024). There was no association between the frequency of red meat, poultry, fish, shrimp, soy products, milk, vegetables, and egg intakes and the risk of precocious puberty after adjusting for potential confounders. Besides, no statistical significance was found in the frequency of fried foods and soft drinks between the two groups (Table S2).

### Amount of dietary intake and precocious puberty risk

**Fig. 2:**
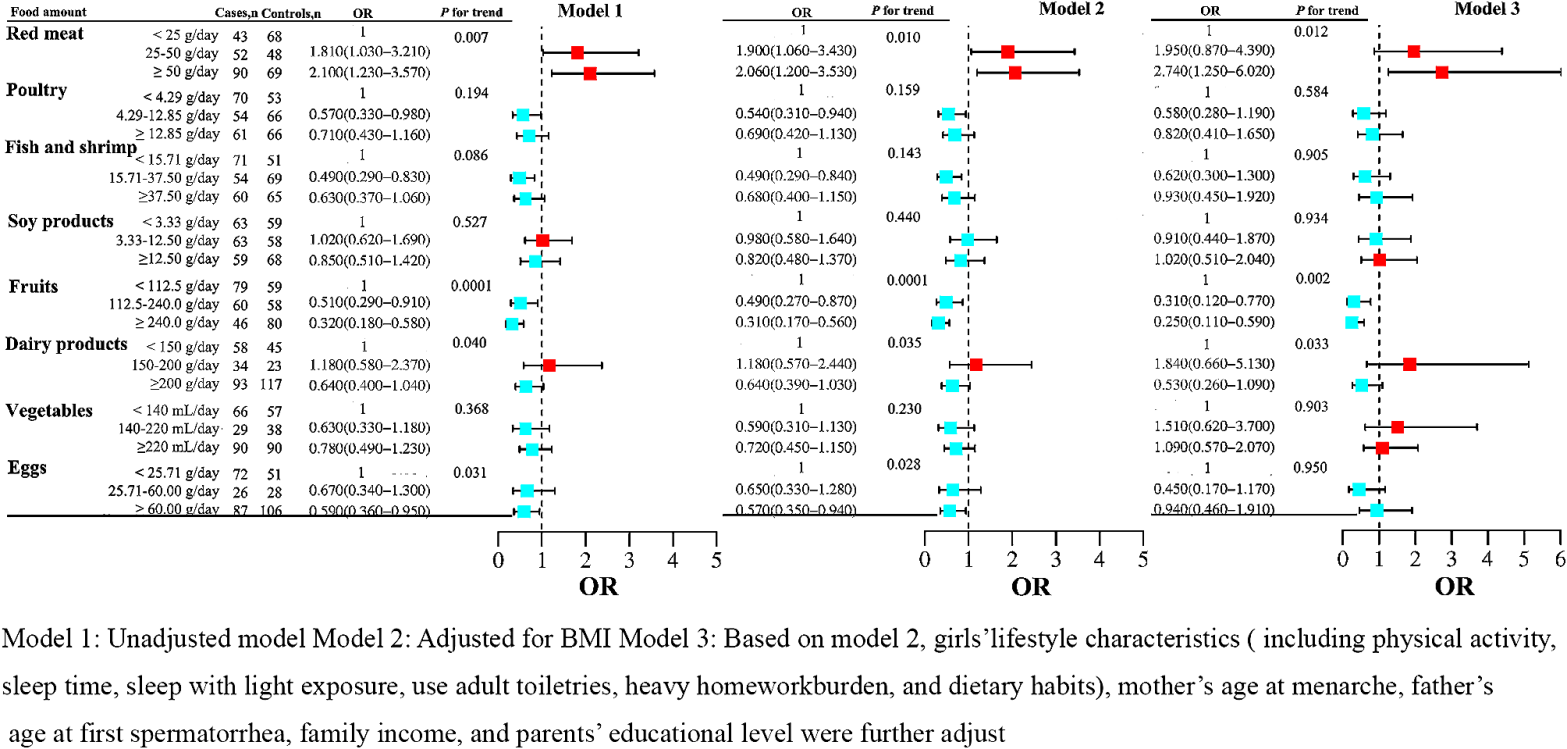
Odds ratios for precocious puberty risk across the amount of dietary intake

Conditional logistic regression models were used to explore the amount of dietary intake during precocious puberty (Fig. 2).

In model 3, after multivariate adjustment, a higher amount of red meat intake was associated with an increased risk of precocious puberty (*P* for trend = 0.012). Specifically, compared with consuming < 25 g/day of red meat, the adjusted ORs (CI) for the risk of precocious puberty were 1.95 (0.87, 4.39), and 2.74 (1.25, 6.02), respectively when consuming 25–50 g/day and 50 g/day red meat.

Also in model 3, a higher number of vegetables and fruits intake was inversely associated with precocious puberty risk (vegetables: *P* for trend = 0.002; fruits: *P* for trend = 0.033). Compared with consuming < 112.5 g/day vegetables, the adjusted ORs (CI) for the risk of precocious puberty were 0.31 (0.12, 0.77), and 0.25 (0.11, 0.59), respectively when consuming 112.5-240.0 g/day and $\ge$240.0 g/day vegetables. Furthermore, when comparing the amount of fruit intake $\ge$200 g/day with <150 g/day, the adjusted OR (95% CI) was 0.53 (0.26, 1.09). Notably, the amount of fish, shrimp, soy products, milk, and egg intake had no significant effect on the risk of precocious puberty, after multivariate adjustment.

### Dietary patterns results

The factor loading values of 13.73%, 11.64%, 11.41%, and 9.03% collectively accounted for 45.81% of the total variation in Table 3. The first dietary pattern (factor 1), referred to as the “high calorie diet”, exhibited a strong positive loading for fried foods and soft drinks. The second dietary pattern (factor 2), termed the “high vegetable and protein diet”, displayed a high loading for vegetables, eggs, and soy products. The third dietary pattern (factor 3) was identified as the “traditional diet”, which indicated the highest positive loading for poultry, fish and shrimp, vegetables, fruits, and dairy products. The forth dietary pattern (factor 4) was called the “high animal food and fruits diet”. This pattern exhibited the highest positive loading for red meat, eggs, and fruits.

**Table 3:** Dietary pattern factor load array(*n* = 370)

Dietary pattern	Food composition	Factor load factor	Variance devoting rates	Cumulative variance contribution rate
High calorie dietary pattern	fried foods and soft drinks	0.51–0.67	0.14	0.13
High vegetable and protein dietary pattern	vegetables, eggs, and soy products	0.55–0.69	0.12	0.25
Traditional dietary pattern	poultry, fish and shrimp,vegetables, fruits, and dairy products	0.34–0.72	0.11	0.37
High animal food and fruits dietary pattern	red meat, fruits, and eggs	0.30–0.81	0.09	0.46

There was a statistically significant difference between the two groups in high calorie dietary pattern and high animal food and fruits dietary pattern, while it was not statistically significant in terms of high vegetable and protein dietary pattern and traditional dietary pattern in Table 4.

**Table 4:** Comparison of different dietary pattern between the case group and control group

Dietary pattern	Cases (*n* = 185)	Controls (*n* = 185)	χ²	*P* value
High calorie dietary pattern, N(%)				
Q1	57(30.81)	36(19.46)	10.47	0.02
Q2	47(25.41)	45(24.32)		
Q3	35(18.92)	58(31.35)		
Q4	46(24.86)	46(24.86)		
High vegetable and protein dietary pattern, N(%)				
Q1	38(20.54)	55(29.73)	5.74	0.13
Q2	48(25.95)	44(23.78)		
Q3	54(29.19)	39(21.08)		
Q4	45(24.32)	47(25.41)		
Traditional dietary pattern, N(%)				
Q1	44(23.78)	49(26.49)	0.67	0.88
Q2	46(24.86)	46(24.86)		
Q3	46(24.86)	47(25.41)		
Q4	49(26.48)	43(23.24)		
High animal food and fruits dietary pattern, N(%)				
Q1	50(27.03)	43(23.24)	8.67	0.03
Q2	55(29.73)	37(20.00)		
Q3	44(23.78)	49(26.48)		
Q4	36(19.46)	56(30.27)		

After adjusting for age and BMI, logistic regression analysis revealed that the “high vegetable and protein dietary pattern” (OR = 0.78, 95% CI: 0.63–0.97) was identified as a protective factor for precocious puberty, while the “high animal food and fruits dietary pattern” (OR = 1.36, 95% CI: 1.09–1.69) was considered as a risk factor.

## Discussion

The present study compared the dietary intake between 185 precocious puberty girls and 185 age-matched controls. The association between the frequency and amount of dietary intake and the risk of precocious puberty was examined. Several important findings were derived. To the best of our knowledge, this is the first study examining the relationship between seven food groups recommended by the dietary guidelines for Chinese school-aged children (2022) and precocious puberty in Chinese girls.

This study for the first time showed that the intake of poultry and red meat of the children in the case group and the control group was higher than the recommended dietary intake, while the intake of vegetables and dairy products was lower than the recommended dietary intake. These results suggested the unreasonable dietary structure of the control and case groups. Both groups needed to increase the intake of vegetables and dairy products and reduce poultry and red meat in order to achieve a rational diet and improve nutritional health status. Moreover, the vegetable intake in the case group was significantly lower than that of the control group. These results indicate that the current dietary structure of children is not reasonable enough, especially for girls with precocious puberty.

The high animal food and fruits dietary pattern, heavy in red meat, fruits, and eggs, was found to be significantly positively associated with precocious puberty after adjustment for age and BMI. This study also revealed that a higher amount of red meat intake was associated with a greater risk of developing precocious puberty in girls. A recent cohort study showed that increased intake of meat by girls during childhood was related to early-age menarche, which was consistent with our findings [36]. Additionally, girls who consumed red meat 2 times or more per day were 64% more likely to develop early-age menarche [36]. Several mechanisms may explain how red meat consumption affects precocious puberty. For instance, micronutrients, such as iron and zinc, in red meat, may signal the onset of puberty in the body, as these nutrients are essential to sustain pregnancy and the survival and development of offspring. A small randomized trial reported that zinc supplementation led to an earlier age of menarche [36]. Another mechanism may be through the development of adiposity. Our study found that the obesity rate of the case group was higher than that of the control group, which reflected that obesity was a risk factor for precocious puberty and consistent with the previous research findings [37–39]. Childhood obesity is associated with early menarche [40], and the intake of some food (including hamburgers and hot dogs) in the red meat group was associated with children being overweight in this population [41]. Additionally, previous studies found that increased intake of animal protein may lead to early puberty [4, 42]. This may be partially due to the protein-mediated secretion of the Insulin-like Growth Factor I (IGF-1). Increased intake of animal protein could potentially promote the secretion of IGF-1 [43]. IGF-I might regulate the reproductive system via widespread effects on the hypothalamus, pituitary, and ovaries through its endocrine, paracrine, and autocrine actions based on the developmental and hormonal state [44, 45]. Moreover, animal models showed that IGF-1 could promote the production of GnRH [46]. Therefore, the higher intake of red meat may increase the secretion of IGF-1, which in turn increases the risk of precocious puberty. Moreover, high fat content in red meat may contribute too as previous studies have reported that a high dietary fat intake led to early onset of pubertal growth in children [4, 47].

The high vegetable and protein diet pattern was identified as a protective factor for precocious puberty. Additionally, our study found that a higher frequency of daily fruit and vegetable intake was associated with a lower risk of precocious puberty in girls. One study has suggested that dietary fiber may reduce the body’s estrogen levels by inhibiting the dissociation of estrogen conjugates and increasing fecal excretion of estrogen, thereby delaying pubertal development [12]. Furthermore, Koo et al. reported that the fiber intake in the highest quartile (= 25.5 g/day) led to a 0.54-fold reduction in the risk of early menarche in 637 Canadian girls age 6–14 years compared to fiber intake in the lowest quartile (≤ 18.2 g/day) [48]. Similarly, Tian et al. showed that children and adolescents who had a higher dietary fiber intake, especially those derived from fruits, had a later onset of adolescence [49]. However, “high animal food and fruits dietary pattern” was considered as a risk factor in our study. This phenomenon may be attributed to the high content of monosaccharide in fruits, which can result in an excessive intake of monosaccharide and subsequently lead to the occurrence of obesity. The research conducted by Wang et al. indicates that an increased consumption of fruits may pose a risk for childhood overweight and obesity [50]. Consequently, it is advised that children limit their fruit consumption and adhere to dietary guidelines regarding fruit intake. Furthermore, future cohort studies with larger sample sizes are necessary to further investigate the association between fruit consumption and precocious puberty.

In the present study, we found that, compared with unadjusted regression analyses, the associations between the frequency of poultry, fish, shrimp, soy products, dairy products, fruits and vegetables intakes and risk of precocious puberty did not change markedly after adjusting for BMI in the logistic regression analyses (Fig. 1). In addition, compared with unadjusted Model 1, no significant change was found in the associations between the number of poultry, fish, shrimp, soy products, and dairy products intakes and the risk of precocious puberty after adjusting for BMI in Model 2 (Fig. 2). Eggs in high animal food and fruits dietary pattern were considered as a risk factor, while they were identified as a protective factor in high vegetable and protein diet pattern. The effect of egg intake on precocious puberty has not been reported in previous studies. We recommend girls to eat the number of eggs according to dietary guidelines for chinese school-aged children [21]. As a result of the small sample size, additional studies are required to further investigate the relationship between egg intakes and precocious puberty.

Soy products were identified as a protective factor in high vegetable and protein diet pattern in our study. The impact of soy products on adolescence is currently a subject of intense debate. A longitudinal study indicated that excessive consumption of soy products may lead to a delay in the onset of puberty [15]. However, a systematic review and meta-analysis showed a nonsignificant association between soy intake and earlier menarche [51]. These conflicting findings may be attributed to issues such as sample representativeness, research analysis methodologies, or the presence of an unclear mechanism underlying the relationship between soy products and precocious puberty. In the future, more large-sample and high-quality studies are needed to explore the relationship between soy products and precocious puberty.

There was a statistically significant difference between the two groups in high calorie dietary pattern. This dietary pattern was found to potentially influence the timing of puberty through three main factors: high fat intake, high sugar consumption, and obesity resulting from excessive calorie intake. The consumption of fried foods, in particular, has been strongly linked to obesity and rapid weight gain, which may serve as a predictive factor for an earlier onset of menarche and other indicators of puberty [52]. Nevertheless, upon conducting logistic regression analysis and adjusting for age and BMI, no significant association was found between a high calorie dietary pattern and precocious puberty in our study. This may be due to the small sample size of this study. Besides, we only analyzed the frequency of fried foods and soft drinks, while the intake of fried foods and soft drinks was not collected for analysis. Furthermore, previous research has demonstrated inconclusive findings regarding the association between overall energy and fat intake and the occurrence of early puberty in the general population. For instance, one study discovered an inverse relationship between childhood fat intake and age at the onset of puberty [53], while another study proposed no significant correlation between total fat intake and puberty [47]. A cohort study conducted in the UK demonstrated that there is a correlation between higher consumption of polyunsaturated fatty acids during early to mid-childhood and an earlier onset of menarche [47]. Moreover, a systematic review revealed that increased intake of polyunsaturated fatty acids is also linked to a heightened risk of premature menarche, while monounsaturated fatty acids have the opposite effect [54]. Further large-scale population surveys are required to examine the potential impact of the frequency and consumption of fatty and sugary beverages on the initiation of puberty, as well as the association between the intake of different types of fatty acids and puberty.

In addition to dietary factors, this study also found a higher proportion of children in the case group sleeping with light exposure, which may be one of the risk factors for precocious puberty. The findings of Crowley et al. study indicated support for a greater sensitivity to evening light in early pubertal children [55]. The increased sensitivity to light in younger adolescents suggested that exposure to evening light could be particularly disruptive to sleep regulation for this group [55]. Another research reported that exposure to light at night can inhibit the secretion of melatonin in the body, which can reduce the level of circulating estrogen in the body [56, 57], so sleeping with the lights on at night may lead to an increase in the level of circulating estrogen in the body due to a decrease in the secretion of melatonin, thus causing precocious puberty. Animal experiments have also found that light may increase the sensitivity of the hypothalamic-pituitary axis to the positive feedback of estrogen [58, 59], which may be one of the reasons why sleeping with lights on at night causes precocious puberty in children.

Our study found that girls in the precocious puberty group have poorer dietary habits, which may increase their risk of obesity. Childhood obesity increases the rate of early puberty, especially in girls [60]. Recently, increasing attention has focused on the effects of the gut microbiota (GM) on obesity and CPP [61–63]. Dong et al. found that the GM of the idiopathic CPP group was enriched for the microbial functions of cell motility, signal transduction, and environmental adaptation [63]. They speculate that a GM alteration could induce an increase of short-chain fatty acids-producing bacteria, which might up-regulate the expression of leptin and increase the secretion of gonadotrophin-releasing hormone. Eventually, the hypothalamic-pituitary-gonadal axis is activated, and idiopathic CPP occurs [63]. Another research observed that CPP-enriched Parabacteroides positively correlated with luteinizing hormone‐releasing hormone, while serotonin‐producer *Akkermansia* exhibited negative relationships with FSH and LH, which indicated the impact of altered GM on CPP [61].

This study had some strengths. First, it is the first study to investigate the association between precocious puberty and the frequency as well as the amount of specific dietary intake in a paired case-control design. Second, it is the first study to examine the relationship between the seven food groups in the dietary guidelines for chinese school-aged children (2022) and precocious puberty in Chinese girls. Third, we conducted a prospective case-control study design to investigate multiple exposures and their potential association with precocious puberty. This prospective case-control design contributed to recording accurate information and allowed for the exploration of various risk factors by utilizing a comprehensive set of socio-demographic and clinical data, which could not be obtained solely from medical records.

However, a few limitations of the study can be pointed out. First, recall bias may exist due to the nature of the questionnaire survey. Second, dietary intake was only assessed by food frequency questionnaires. Therefore, some dietary intake such as total energy intake or fat intake could not be calculated, thereby limiting the interpretation of the results. In the future, a 3-day food record could be used for an accurate assessment of dietary intake. Third, the cross-sectional design employed in this study precluded the ability to establish a definitive temporal sequence between diet and precocious puberty, thereby hindering the demonstration of a causal relationship between dietary patterns and precocious puberty. Longitudinal studies are needed for addressing this limitation. Moreover, the selection of cases and controls from a single hospital in Shanghai during the study’s design phase introduced a certain degree of selection bias, as the children included in the study were selectively drawn from this specific hospital. Furthermore, it is noteworthy that the study exclusively focused on female subjects, thereby limiting the generalizability of the findings to male individuals. Another shortcoming was that the sample size was not large enough. An increased sample size would increase the power to test the association. Additionally, we acknowledge that the lack of staple food intake was indeed a limitation of this study. Future studies are required to further investigate the relationship between the staple food intake and precocious puberty. Last but not least, GM plays an important role in CPP. We will explore the relationship between GM and CPP in future both human and animal studies.

Exploring the daily dietary behaviour of girls with precocious puberty can help to develop targeted interventions for better tertiary prevention of precocious puberty. This study found that high vegetable and protein diet pattern was a protective factor, while high animal food and fruits dietary pattern was a risk factor for precocious puberty in Shanghainese girls. It is recommended that local policymakers, health caregivers, and parents prioritize the adoption of a well-balanced diet while avoiding girls excessive intake of red meat, eggs, and fruits. Furthermore, efforts should be made to promote children’s dietary diversity, encourage greater vegetable consumption, and assist them in establishing a balanced eating regimen, in order to prevent the occurrence of precocious puberty.

## Conclusion

In conclusion, high vegetable and protein dietary pattern is a protective factor against precocious puberty, while high animal food and fruits dietary pattern is a risk factor for precocious puberty in Chinese girls. The local policymakers, health caregivers, and parents should pay attention to a reasonable intake of red meat, eggs, and fruits in children’s daily diet, increase their intake of vegetables, in order to reduce the risk of precocious puberty.

Large-scale prospective cohort studies are needed in the future to determine the causal relationship between dietary patterns and precocious puberty using more accurate dietary assessment methods to provide further support for public health efforts to guide a balanced diet and better prevent and control precocious puberty.

### Electronic supplementary material

Below is the link to the electronic supplementary material.

Supplementary Material 1

## Data Availability

The original contributions presented in the study are included in the article/supplementary material. Further inquiries can be directed to the corresponding author.

## Abbreviations

CPP: Central Precocious Puberty
PPP: Peripheral Precocious Puberty
HR: Hazard Ratio
FFQ: Food frequency questionnaire
OR: Odds Ratio
