# Supplementary material for: Dietary pattern and precocious puberty risk in Chinese girls: a case-control study
Source: Nutr J. 2024 Jan 31;23:14. doi: 10.1186/s12937-024-00916-6 (PMC10829199; doi:10.1186/s12937-024-00916-6)
Supplement: Supplementary file 1 — Supplementary Material 1 [file 12937_2024_916_MOESM1_ESM.docx]

Table S1. Food Frequency Questionnaire

| Food Name | Eat/drink  or not  1 Yes  0 No | Frequency (answer only one of these) | | | | Average  amount for  each time  (g) |
| --- | --- | --- | --- | --- | --- | --- |
|  |  | Times  per  day | Times  per  week | Times  per  month | Times  per  year |  |
| Red meat |  |  |  |  |  |  |
| Poultry |  |  |  |  |  |  |
| Fish and shrimp |  |  |  |  |  |  |
| Vegetables |  |  |  |  |  |  |
| Fruits |  |  |  |  |  |  |
| Dairy products |  |  |  |  |  |  |
| Eggs |  |  |  |  |  |  |
| Soy products |  |  |  |  |  |  |
| Fried foods |  |  |  |  |  |  |
| Soft drinks |  |  |  |  |  |  |

*Please recall whether you ate these food items in the past 12 months or not, and estimate the frequency and average edible amount.

**Table S2 Odds ratios for precocious puberty risk across the frequency of fried foods and soft drinks**

| **Food frequency** | **Cases,n** | **Controls,n** | **Model 1** | **Model 2** | **Model 3** |  |
| --- | --- | --- | --- | --- | --- | --- |
| **Fried foods** |  |  |  |  |  |  |
| < 1 time/month | 106 | 79 | 1.0 | 1.0 | 1.0 |  |
| 1time/month~1 time/week | 69 | 89 | 0.44 (0.19, 1.01) | 0.45 (0.20, 1.04) | 0.06 (0.00, 37.41) |  |
| ≥ 1 time/week | 10 | 17 | 0.76 (0.33, 1.76) | 0.78 (0.34, 1.83) | 0.21 (0.01, 77.13) |  |
| *P* for trend |  |  | 0.005 | 0.006 | 0.505 |  |
| **Soft drinks** |  |  |  |  |  |  |
| < 1 time/month | 92 | 63 | 1.0 | 1.0 | 1.0 |  |
| 1time/month~1 time/week | 71 | 98 | 0.63 (0.32, 1.22) | 0.63 (0.32, 1.22) | 0.05 (0.00, 33.98) |  |
| ≥ 1 time/week | 22 | 24 | 1.27 (0.66, 2.43) | 1.20 (0.62, 2.31) | 4.82 (0.01, 44.18) |  |
| *P* for trend |  |  | 0.018 | 0.026 | 0.321 |  |

Model 1: Unadjusted model

Model 2: Adjusted for BMI

Model 3: Based on model 2, girls’ lifestyle characteristics (including physical activity, sleep time, sleep with light exposure, use of adult toiletries, heavy homework burden, and dietary habits), mother’s age at menarche, father’s age at first spermatorrhea, family income, and parents’ educational level were further adjusted
